# Bilayer Designed Paper-Based Solar Evaporator for Efficient Seawater Desalination

**DOI:** 10.3390/nano12193487

**Published:** 2022-10-05

**Authors:** Ying Qin, Yongzheng Li, Ruijie Wu, Xiaodi Wang, Jinli Qin, Yingjuan Fu, Menghua Qin, Zhiwei Wang, Yongchao Zhang, Fengshan Zhang

**Affiliations:** 1 Guangxi Key Laboratory of Clean Pulp & Papermaking and Pollution Control, College of Light Industry and Food Engineering, Guangxi University, Nanning 530004, China; 2 State Key Laboratory of Biobased Material and Green Papermaking, Qilu University of Technology, Shandong Academy of Sciences, Jinan 250353, China; 3 Organic Chemistry Laboratory, Taishan University, Taian 271021, China; 4 Huatai Group Co., Ltd., Dongying 257335, China

**Keywords:** bilayered solar evaporator, Fe_3_O_4_ nanoparticles, decorated cellulose fibers, photothermal conversion

## Abstract

Solar desalination devices utilizing sustainable solar energy and the abundant resource of seawater has great potential as a response to global freshwater scarcity. Herein, a bilayered solar evaporator was designed and fabricated utilizing a facile paper sheet forming technology, which was composed of cellulose fibers decorated with Fe_3_O_4_ nanoparticles as the top absorbent layer and the original cellulose fibers as the bottom supporting substrate. The characterization of the cellulose fibers decorated with Fe_3_O_4_ nanoparticles revealed that the in situ formed Fe_3_O_4_ nanoparticles were successfully loaded on the fiber surface and presented a unique rough surface, endowing the absorber layer with highly efficient light absorption and photothermal conversion. Moreover, due to its superhydrophilic property, the cellulose fiber-based bottom substrate conferred ultra-speed water transport capability, which could enable an adequate water supply to combat the water loss caused by continuous evaporation on the top layer. With the advantages mentioned above, our designed bilayered paper-based evaporator achieved an evaporation rate ~1.22 kg m^−2^ h^−1^ within 10 min under 1 sun irradiation, which was much higher than that of original cellulose cardboard. Based on the simple and scalable manufacture process, the bilayered paper-based evaporator may have great potential as a highly efficient photothermal conversion material for real-world desalination applications.

## 1. Introduction

Global freshwater levels are declining dramatically with the development of industrialization and modernization [1,2]. Although water covers 71% of the earth’s surface area, meeting the human demand for freshwater remains a huge challenge as the global freshwater volume is about 2.5% of the total water volume [3,4,5]. To prevent this from happening and address the above-mentioned problems, numerous research efforts have been devoted to the development of low-cost, efficient, and polluted water purification and desalination technologies [6,7]. One of the most prominent desalination methods is the thermal desalination and membrane process [8,9]. However, there are some problems; for example, thermal desalination heat is mainly derived from fossil fuel consumption and dirt is produced [10,11]. The presence of corrosive agents in the membrane process not only increases the cost of desalination, but also reduces the recyclability of the membrane [12]. This also shows the importance and urgency of renewable energy for water desalination [13,14,15].

Solar energy is a sustainable and clean source of energy that is also the source of all energy generation (wind, heat); in addition to heat and power generation, the use of abundant renewable solar energy for desalination is one of the most promising solutions for sustainable clean water production [16,17]. The development of interfacial solar evaporation technology positioned solar conversion and water evaporation at the liquid/vapor interface, while the concepts of ambient energy input and evaporation enthalpy were successively reported to increase the solar thermal efficiency of solar evaporators to over 100% [14,18]. Early designs had a hydrophilic solar absorber layer floating directly on the water surface [13]. This strategy of direct contact with seawater inevitably reduced photothermal conversion and increased heat loss [19,20]. To reduce heat loss, an indirect contact strategy was proposed to isolate the solar absorber layer from the water body [21]. Such a device usually includes a solar absorber and a sponge-like substrate [22]. The upper solar absorber layer is usually composed of photothermal materials, which are broadly classified into plasma metals, carbonaceous materials, conjugated polymers, narrow bandgap semiconductors, and hybrids. Among them, narrow bandgap semiconductor materials such as Ti_2_O_3_, Fe_3_O_4_, and black titanium dioxide have attracted great interest in the field of solar energy utilization [17,23]. When incident light enters, the energy of the photons leads to the production of electron–hole pairs [7]. In turn, these electron–hole pairs can relax to the band edges of the conduction and valence bands, converting the additional energy into heat [24]. Magnetic Fe_3_O_4_ with a bandgap of less than 1.0 eV, which is a typical narrow bandgap semiconductor, has the advantages of low toxicity, low cost, wide solar absorption, and high optical response. Due to their high photothermal conversion capability, Fe_3_O_4_ particles are widely used in medical applications for photothermal therapy [25]. The bottom substrate is usually made of a lightweight, porous, hydrophobic polymer sponge [26]. In contrast, many commercial synthetic polymers break down into microplastics that accumulate in the ecosystem; thus, the environmental impact of many synthetic materials is of great and active concern [27]. To address these issues, the use of lightweight, low-cost, environmentally friendly material platforms for efficient evaporation and other promising functions has become a topic of increasing interest [2,28].

Paper is a fibrous material made from natural plant fibers and processed mechanically, which is a green and sustainable natural polymer [29,30]. Cellulose fibers are generally 1–3 mm in length and are easily interwoven and entangled during the papermaking process, forming fiber flocs. Herein, we reported a facile strategy for desalination by fabricating a blazered paper-based evaporator composed of cellulose fibers decorated with Fe_3_O_4_ nanoparticles as the top absorber layer and the original cellulose fibers as the bottom supporting substrate. The Fe_3_O_4_ nanoparticles decorated cellulose fibers showed that the in situ formed Fe_3_O_4_ nanoparticles loaded on the fiber surface presented a unique rough surface, which endowed the absorber layer with highly efficient light absorption and photothermal conversion. The superhydrophilic cellulose fiber based bottom substrate conferred ultra-speed water transport capability, which could enable an adequate water supply for the water loss caused by continuous evaporation on the top layer. Our designed layered paper-based evaporator achieved an evaporation rate of ~1.22 kg m^−2^ h^−1^ within 10 min under 1 sun irradiation. The as-prepared bilayer designed paper evaporator shows has potential in the fields of desalination and water purification.

## 2. Materials and Methods

### 2.1. Materials

Cellulose fibers (dissolving pulp produced from hardwood) and wet strength agent (polyamide epichlorohydrin, PAE) were obtained from a factory, China. Ferric chloride hexahydrate (FeCl_3_∙6H_2_O) and trisodium citrate dihydrate were purchased from Sinopharm Chemical Reagents Co., Ltd. Ferrous chloride tetrahydrate (FeCl_2_∙4H_2_O) was purchased from Tianjin Danao Chemical Reagent Factory. Ammonia (NH_3_∙3H_2_O) was purchased from Kunshan Jincheng Reagent Co., Ltd. All chemicals were of analytical grade and used without further purification.

### 2.2. Preparation of Fe_3_O_4_-Decorated Fibers

In this study, Fe_3_O_4_-decorated fibers were prepared by a typical in situ synthesis process. Cellulose fibers were dispersed in deionized water, and 5% wet strength agent (based on oven-dry dissolved pulp) was added to prepare 6% fiber suspension. Meanwhile, FeCl_3_∙6H_2_O (4.3350 g) and FeCl_2_∙4H_2_O (1.9900 g) were well mixed in 50 mL deionized water to prepare solution A. Then, 200 g (12 g oven-dry cellulose fibers) fiber suspension was added to 500 mL 1 mol/L ammonia. Then, solution A was added into the suspension, placed in a thermostatic water bath at 70 °C, and stirred for 30 min. After the reaction, trisodium citrate dihydrate was added to the suspension, and reacted in a thermostatic water bath at 40 °C for 2 h. Finally, after the suspension was allowed to stand for 6 h, the supernatant was removed, and the Fe_3_O_4_-decorated fibers was prepared.

### 2.3. Preparation of Bilayered Paper-Based Evaporator

The bilayered paper-based evaporator consisted of two layers. The first layer was made of Fe_3_O_4_-decorated fibers by vacuum filtration; on the basis of the first layer, the second layer was prepared by further vacuum filtration of the unmodified cellulose fiber suspension, thus preparing a blazered paper-based evaporator. Then, 0.5 wt.% (based on oven-dry cellulose fiber) wet strength agent was added to the fiber suspension before vacuum filtration. The mass ratio of Fe_3_O_4_-decorated fibers to fiber suspension was 1:2 (based on oven-dry cellulose fibers). The prepared blazered paper-based evaporator was dried in a drying oven at 45 °C for 6 h.

### 2.4. Solar Steam Generation Test and Desalination Test

Solar steam generation and desalination tests were conducted at room temperature (25 °C) and humidity (32%) using a solar simulator (CEL-HXF300-T3, CEAULIGHT, Beijing, China) and an electronic balance connected to a computer. The solar evaporator of an appropriate size was placed into a water container (diameter 15 mm, height 100 mm). The light intensity was calibrated by a photo radiometer (PL-MW2000, Perfect Light, Beijing, China), and it was maintained at 1 kW/m^2^ (1 sun illumination). The temperature of the top surface of the evaporator and the weight change of the water container were measured in real-time by infrared thermal imager and analytical balance respectively. A 3.5 wt% NaCl solution was used to simulate seawater. The calculation of energy conversion efficiency referred to the previous literature [31].

### 2.5. Characterization of Paper-Based Evaporator

The morphology and internal microstructure of the bilayered paper-based evaporator were observed by scanning electron microscope (SEM, Hitachi Regulus8220, Hitachi, Ltd., Tokyo, Japan) and transmission electron microscope (TEM, JEM 2100, JEOL Ltd., Tokyo, Japan). The ultraviolet–visible-near infrared spectrophotometer (UV-Vis-NIR, Agilent Cary 5000, Agilent Technologies Inc, Santa Clara, CA, USA) was recorded in the range 200–2500 nm. Fourier transform infrared (FTIR, Bruker Alpha, Bruker Optics Inc., Karlsruhe, Germany) spectrometer was used to characterize the surface structural groups. X-ray photoelectron spectroscopy (XPS, Thermo ESCALAB250Xi, Thermo Fisher Scientific, Waltham, MA, USA) was used to characterize the surface element composition. The chemical composition and morphology were analyzed by X-ray diffraction (XRD, SmartLab SE, Rigaku Smart Lab SE, Rigaku Corporation, Tokyo, Japan). The scanning range was 2θ = 10~80°.

## 3. Results and Discussion

### 3.1. Preparation of Bilayered Paper-Based Evaporator

**Figure 1:**
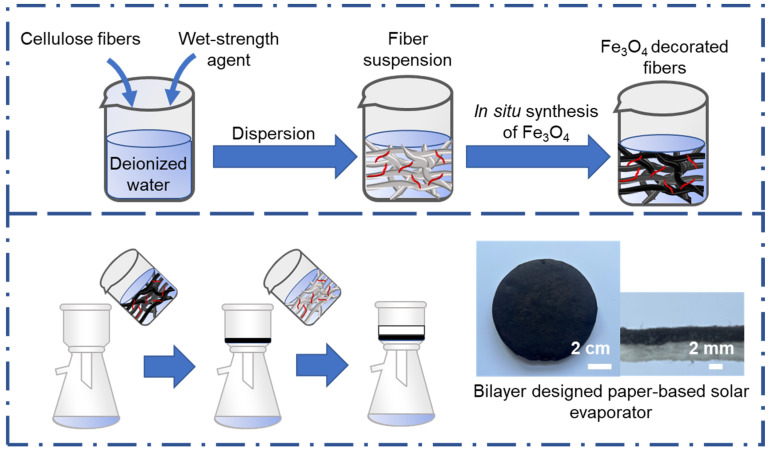
Schematic illustration for the fabrication of the bilayer designed paper-based solar evaporator.

The preparation process of the bilayer designed paper-based solar desalination device is shown in Figure 1. Firstly, cellulose fibers were decorated with the in situ formed Fe_3_O_4_ nanoparticles, and a black suspension was thus prepared. Secondly, the Fe_3_O_4_ nanoparticles-decorated fiber was filtered into cardboard that was covered with original cellulose fibers. The bilayer designed cardboard was fabricated and had two different colors. The excellent photothermal conversion capability of Fe_3_O_4_ nanoparticles has recently been demonstrated as a solar light absorber [32,33]. Moreover, the natural hydrophilic cellulose fibers possess water retention capacity [34]. Therefore, the fabricated bilayer cardboard was considered as a solar desalination device. The top layer, consisting of black Fe_3_O_4_ nanoparticles-decorated cellulose fibers, absorbs sunlight and transforms it to thermal energy, which heats surface water to generate steam. The bottom layer, composed of original cellulose fibers, could continuously transfer water from bottom to top surface as a supply to compensate for the loss of evaporated water.

### 3.2. Characterization of Bilayered Paper-Based Evaporator

**Figure 2:**
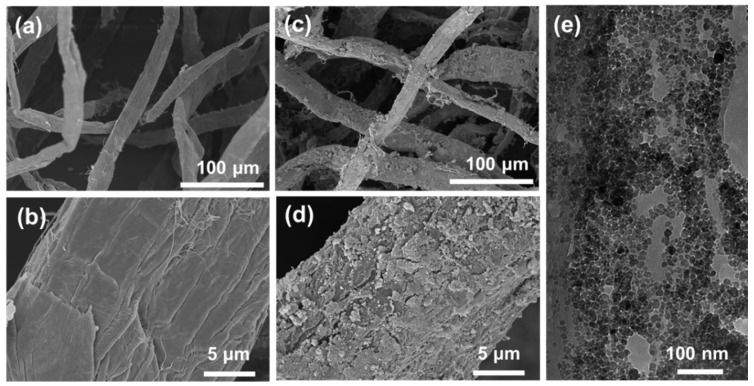
SEM images showing cellulose fibers (**a**,**b**) and Fe_3_O_4_ nanoparticles-decorated cellulose fibers (**c**,**d**); TEM image of the in situ formed Fe_3_O_4_ nanoparticles on the fibers surface (**e**).

The morphology and microstructure of cellulose fibers and Fe_3_O_4_ nanoparticles decorated cellulose fibers were observed by scanning electron microscopy (SEM). As shown in Figure 2, the cellulose fibers were about 30–40 μm in diameter and exhibited relatively smooth and bright surface morphology (Figure 2a,b). After in situ loading of Fe_3_O_4_ nanoparticles, the rough cellulose fibers surface and the deep narrow uneven valley morphology were observed, demonstrating that cellulose fibers were successfully decorated with Fe_3_O_4_ nanoparticles that covered the entire fiber surface (Figure 2c,d). The unique rough surface could contribute to enhance the light absorbability of the Fe_3_O_4_ nanoparticles-decorated cellulose fibers by extending optical path of multiple scattering and reducing reflectance. By contrast, the untreated cellulose fibers could sustain relatively high light reflectance due to their bright and smooth surface, thus greatly reducing the absorption of sunlight. Moreover, transmission electron microscopy (TEM) was applied to further reveal the morphology and microstructure of the formed Fe_3_O_4_ nanoparticles on the cellulose fiber surface. It was clear observed that the formed Fe_3_O_4_ nanoparticles had an average particle size around 10–20 nm and self-assembled a nanoscale layer on the cellulose fibers surface (Figure 2e), which was consistent with the surface color observed from the digital image (Figure 1).

**Figure 3:**
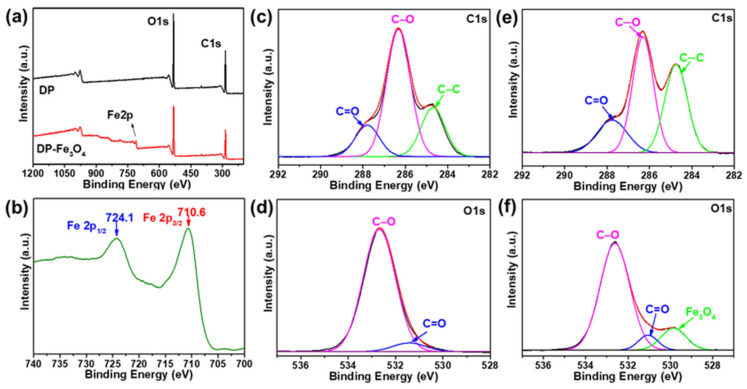
XPS spectra of cellulose fibers (**a**) and Fe_3_O_4_ nanoparticles-decorated cellulose fibers (**a**,**b**); (**c**) C1s and (**d**) O1s XPS spectra of cellulose fibers; (**e**) C1s and (**f**) O1s XPS spectra of Fe_3_O_4_ nanoparticles-decorated cellulose fibers.

X-ray photoelectron spectroscopy (XPS) analysis was performed to determine the elemental composition and oxidation states. As shown in Figure 3a, both samples exhibited clear peaks at 286.0 and 533.0 eV attributable to C 1s and O 1s, respectively. Remarkably, compared with cellulose fibers, two peaks could be clearly observed in the Fe 2p region of the XPS spectrum of Fe_3_O_4_ nanoparticles-decorated cellulose fibers, including the peak positions of Fe 2p 3/2 (710.6 eV) and Fe 2p 1/2 (724.1 eV), which are consistent with the f values reported in the literature for Fe_3_O_4_ [35]. This result demonstrated the formation of Fe_3_O_4_ on the decorated cellulose fibers. In the comparison of the high-resolution C 1s spectra, both samples contained C 1s peaks ascribed to C–C (284.69 eV), C–O (286.33 eV) and C=O (287.80 eV). However, after in situ formation of Fe_3_O_4_ nanoparticles, a decrease in surface C–O bonding was detected, indicating that Fe_3_O_4_ nanoparticles were grown on the surface of cellulose fibers (Figure 3c–e). Interestingly, the high-resolution O 1s spectra of Fe_3_O_4_ nanoparticles-decorated cellulose fibers showed a significant transition peak compared to the original cellulose fibers (Figure 3d,f), which should give rise to a large number of hydrogen bonding interactions between hydroxyl groups on the surface of the in situ grown ferric tetroxide clusters and exposed hydroxyl groups of cellulose fibers.

**Figure 4:**
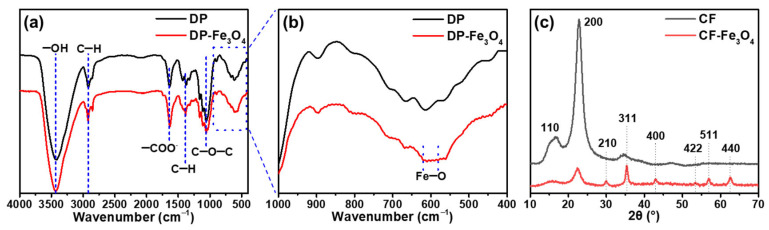
FTIR spectra (**a**,**b**) and XRD spectrum (**c**) of cellulose fibers and Fe_3_O_4_ nanoparticles-decorated cellulose fibers.

As shown in Figure 4a, Fourier transform infrared (FTIR) spectroscopy of Fe_3_O_4_ nanoparticles-decorated cellulose fibers and the original cellulose fibers presented the peaks at 2918 and 1374 cm^−1^ corresponding to the C–H stretching vibration of cellulose [36]. Two weak peaks at 580 and 620 cm^−1^ assigned to the stretching vibration of the Fe-O bond in Fe_3_O_4_ appeared in Fe_3_O_4_ nanoparticles-decorated cellulose fibers, indicating that the in situ formed Fe_3_O_4_ nanoparticles were loaded on the cellulose matrix (Figure 4b) [37,38]. X-ray diffraction (XRD) patterns revealed the phases of cellulose fibers and Fe_3_O_4_ nanoparticles-decorated cellulose fibers (Figure 4c). The two typical peaks at 16.4° and 22.6° could be clearly observed in both two samples, which were attributed to the (110) and (200) planes of cellulose, respectively, demonstrating their crystal structure [39]. Moreover, the cellulose diffraction peaks in the Fe_3_O_4_ nanoparticles-decorated cellulose fibers weakened significantly compared to the original cellulose fibers, suggesting that the crystallinity of cellulose in the decorated cellulose fibers has been affected by the Fe_3_O_4_ nanoparticles loaded on the cellulose fibers. Upon in situ formation of Fe_3_O_4_ layer on the cellulose surface, the emerged Fe_3_O_4_ nanoparticles-decorated cellulose fibers the distinct diffraction peaks representing Fe_3_O_4_ at 30.0, 35.4, 43.1, 53.3, 56.9, and 62.5°, emerged, which were assigned to the (210), (311), (400), (422), (511), and (440) planes of cubic Fe_3_O_4_ [40,41]. This was further confirmed in situ formation of Fe_3_O_4_ nanoparticles on the cellulose fibers.

### 3.3. Photothermal Conversion of Bilayered Paper-Based Evaporator

**Figure 5:**
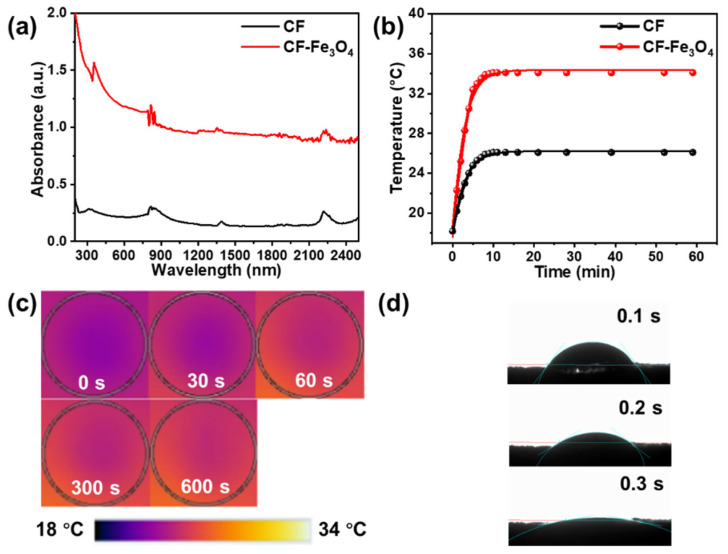
(**a**) The light absorption spectra of the Fe_3_O_4_ nanoparticles-decorated paper-based evaporator and original cellulose cardboard. (**b**) Time-dependent average temperature of the top absorber surface. (**c**) IR images of the wet Fe_3_O_4_ nanoparticles-decorated paper-based evaporator and original cellulose cardboard under 1 sun irradiation. (**d**) Time-lapse snapshots of absorption of a water droplet by the Fe_3_O_4_ nanoparticles-decorated paper-based evaporator.

The Fe_3_O_4_ nanoparticle-based light absorber has demonstrated outstanding solar absorption efficiency in the full solar spectrum, which is a critical factor for achieving highly-efficient photothermal conversion and high evaporation efficiency [5,17,42,43]. The photothermal conversion property of the fabricated bilayered paper-based evaporator was evaluated. The original cellulose cardboard was used as a reference. The light absorption of both the fabricated bilayered paper-based evaporator and original cellulose cardboard was measured by the UV/vis–NIR spectrophotometer in the wavelength range from 200 to 2500 nm. Compared to the original cellulose cardboard, the bilayered paper-based evaporator exhibited a much higher light absorption (1.0–2.0) in both the UV–visible region and the near-infrared region, which can be attributed to the broad absorption spectrum of Fe_3_O_4_ nanoparticles as well as the enhanced light scattering and reduced reflectance from the unique rough surface of the Fe_3_O_4_ nanoparticles-decorated cellulose fibers. The excellent light absorption indicated the great potential of the bilayered paper-based evaporator for highly efficient solar desalination. As shown in Figure 5b,c, under 1 sun irradiation, the surface temperature of the fabricated bilayered paper-based evaporator rapidly increased from water temperature to the equilibrium temperature (34.4 °C) within 10 min when the evaporative cooling of water and the absorption heating of the surface to reach the energy equilibrium state. In contrast, the temperature of the original cellulose cardboard at equilibrium state was maintained basically below 26.2 °C. The results further confirmed the efficient energy-harvesting ability and outstanding photothermal conversion capability of the absorber layer consisting of the Fe_3_O_4_ nanoparticles-decorated cellulose fibers, which significantly improved the desalination performance by continuously outputting thermal energy to drive the heating and evaporation of the water body. As shown in Figure 5d, a water droplet could rapidly disappear into the top Fe_3_O_4_ nanoparticles-decorated cellulose layer of the fabricated bilayered paper-based evaporator within 0.3 s, demonstrating the superhydrophilicity property of the Fe_3_O_4_ nanoparticles decorated cellulose fibers. During the solar evaporation process, the bottom water can be quickly pumped from bulk water to the top evaporation layer to replace the water loss due to continuous solar evaporation.

### 3.4. Solar Desalination Performance

**Figure 6:**
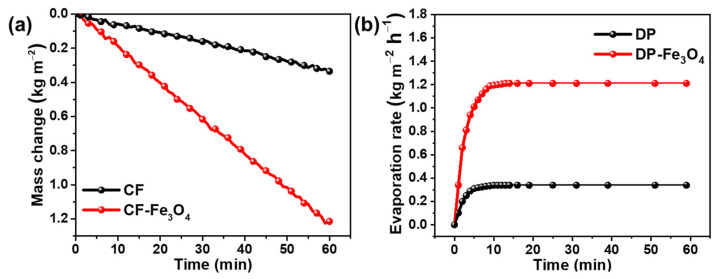
Mass loss of water (**a**) and evaporation rate (**b**) in the simulated seawater solution for the Fe_3_O_4_ nanoparticles-decorated paper-based evaporator and original cellulose cardboard under 1 sun illumination.

The solar vapor generation performance of the fabricated bilayered paper-based evaporator was investigated under 1 sun illumination. The fabricated evaporator was directly placed on the bulk water surface and the weight change in water was recorded in real-time upon light illumination. When the simulated sun light irradiated the evaporator, the absorber layer, consisting of the Fe_3_O_4_ nanoparticles-decorated cellulose fibers, enabled rapid light absorption and photothermal conversion, and at the same time the water started to evaporate. Compared to original cellulose cardboard, the mass loss of the fabricated bilayered paper-based evaporator versus irradiation time presented a more linear relationship and a larger mass loss, indicating better solar evaporation performance (Figure 6a). As shown in Figure 6b, under 1 sun illumination, the evaporation rate of the fabricated bilayered paper-based evaporator reached ~1.22 kg m^−2^ h^−1^ in 10 min, which was much higher than that of original cellulose cardboard. This result was consistent with temperature changes on the absorber surface of the fabricated evaporator. The excellent performance of the fabricated bilayered paper-based evaporator can be attributed to the following synergistic effects: (1) the top evaporation layer loaded with numerous in situ formed Fe_3_O_4_ nanoparticles that present a unique rough surface for highly efficient light absorption and photothermal conversion; (2) the ultra-speed water transport capability of cellulose based bottom substrate enabled an adequate water supply to replace the water loss caused by continuous evaporation on the top layer. More importantly, previously reported solar desalination devices produced using cellulose or nanocellulose often entail complex fabrication processes and high material cost, which restrict the widespread application of these novel technologies. Our designed bilayered paper-based evaporator prepared by a facile method offers a scalable, environmentally friendly, and low-cost seawater desalination platform for practical applications in water purification [44,45,46,47]. Therefore, the outstanding steam evaporation of the fabricated bilayered paper-based evaporator makes it an ideal photothermal device in the field of solar-driven desalination and water purification.

## 4. Conclusions

In summary, we have demonstrated a simple and scalable approach for the fabricating bilayered solar evaporator with Fe_3_O_4_ nanoparticles decorated cellulose fibers as the top absorber layer and the original cellulose fibers as the bottom supporting substrate. After loading Fe_3_O_4_ nanoparticles of cellulose fibers, the top evaporation layer possessed a unique rough surface, which endow the absorber layer with highly efficient light absorption and photothermal conversion. Owing to the natural super-hydrophilic property, the cellulose fibers based bottom substrate rendered the ultra-speed water transport capability, which could enable an adequate water supply for the water loss caused by continuous evaporation on the top layer. Considering the irreplaceable advantages, the as-designed bilayered paper-based evaporator possessed an evaporation rate ~1.22 kg m^−2^ h^−1^ within 10 min under 1 sun irradiation. The demonstrated highly efficient solar evaporation device allow significant opportunities for the practical application of cost-effective, easy-to-manufacture desalination and water purification technology.

## Data Availability

The data presented in this study are available on request from the corresponding authors.
